# Tumour necrosis factor activates the mitogen-activated protein kinases p38α and ERK in the synovial membrane *in vivo*

**DOI:** 10.1186/ar1797

**Published:** 2005-07-28

**Authors:** Birgit Görtz, Silvia Hayer, Birgit Tuerck, Jochen Zwerina, Josef S Smolen, Georg Schett

**Affiliations:** 1 Division of Rheumatology, Department of Internal Medicine III, University of Vienna, Vienna, Austria; 2 Institute of Pathology, University of Giessen, Giessen, Germany

## Abstract

Tumour necrosis factor (TNF) is considered to be a major factor in chronic synovial inflammation and is an inducer of mitogen-activated protein kinase (MAPK) signalling. In the present study we investigated the ability of TNF to activate MAPKs in the synovial membrane *in vivo*. We studied human TNF transgenic mice – an *in vivo *model of TNF-induced arthritis – to examine phosphorylation of extracellular signal-regulated kinase (ERK), c-Jun amino terminal kinase (JNK) and p38MAPKα in the inflamed joints by means of immunoblot and immunohistochemistry. In addition, the effects of systemic blockade of TNF, IL-1 and receptor activator of nuclear factor-κB (RANK) ligand on the activation of MAPKs were assessed. *In vivo*, overexpression of TNF induced activation of p38MAPKα and ERK in the synovial membrane, whereas activation of JNK was less pronounced and rarely observed on immunohistochemical analysis. Activated p38MAPKα was predominantly found in synovial macrophages, whereas ERK activation was present in both synovial macrophages and fibroblasts. T and B lymphocytes did not exhibit major activation of any of the three MAPKs. Systemic blockade of TNF reduced activation of p38MAPKα and ERK, whereas inhibition of IL-1 only affected p38MAPKα and blockade of RANK ligand did not result in any decrease in MAPK activation in the synovial membrane. These data indicate that TNF preferentially activates p38MAPKα and ERK in synovial membrane exposed to TNF. This not only suggests that targeted inhibition of p38MAPKα and ERK is a feasible strategy for blocking TNF-mediated effects on joints, but it also shows that even currently available methods to block TNF effectively reduce activation of these two MAPKs.

## Introduction

Chronic inflammation of the synovial membrane (synovitis) is a hallmark of rheumatoid arthritis (RA). This process is fueled by proinflammatory cytokines, which not only induce but also maintain synovitis and therefore play an important role in progressive joint destruction [1,2]. Several cytokines are currently considered to be key molecules in joint inflammation, but the evidence that tumour necrosis factor (TNF) is crucial to development of chronic destructive arthritis is most compelling. This is primarily supported by the clinical efficacy of TNF blocking agents in the treatment of RA but also by the fact that overexpression of TNF is sufficient to cause inflammatory arthritis in mice [3-7]. In addition, expression of TNF has been detected in the synovial membrane of RA patients, and cultivated cells from the synovial tissue produce increased amounts of TNF [8-10].

The effects of TNF-α are mediated via a complex network of signalling pathways. Apart from activation of nuclear factor-κB, many signals are transduced through mitogen-activated protein kinases (MAPKs), which include extracellular signal-regulated kinase (ERK), c-Jun amino-terminal kinases (JNK) and p38MAPKα [11]. These molecules mediate activation of many key transcription factors, such as the activator protein-1 complex, which then facilitates induction and transcription of the relevant proinflammatory genes, such as cytokines, chemokines and matrix metalloproteinases [12]. Indeed, these structures are considered to be promising therapeutic targets, and several small molecule based inhibitors are currently being tested for their antiarthritogenic potential [13-16].

It is currently unclear whether TNF equally activates all three MAPK families in arthritis or has a certain predilection toward activating one of the families in the synovial tissue *in vivo*. Despite the potential of TNF to activate all three MAPKs, the pathways that are of relevance to chronic destructive arthritis remain to be elucidated. However, if we are to design therapeutic tools that can effectively block TNF-mediated inflammatory responses, then we must define the major signalling targets of TNF in inflammatory joint disease *in vivo*. Interestingly, all three MAPK families – p38MAPKα, ERK and JNK – are activated in RA synovial membrane, and TNF-α has the potential to signal through all of them [17,18]. Therefore, each of these different MAPKs is a potential therapeutic target.

In the present study we investigated the effect of *in vivo *overexpression of TNF on MAPK signalling in synovial tissue. Mice transgenic for human TNF (hTNFtg mice), which develop a chronic inflammatory joint disease, were assessed for immunohistochemical evidence of activation of the three MAPK families (ERK, JNK and p38MAPKα). Moreover, we defined the cell types in the synovial membrane that exhibit MAPK activation and investigated the effects of anticytokine therapies on MAPK signalling.

## Materials and methods

### Animals and treatments

Heterozygous Tg197 human TNF-α transgenic (hTNFtg) mice (strain C57/Bl6), which develop a chronic inflammatory and destructive polyarthritis within 4–6 weeks after birth, were described previously [7]. We investigated five groups of hTNFtg mice aged 10 weeks, of which one group was left untreated. Of the other four groups one was treated with anti-TNF (infliximab; Centocor, Leiden, The Netherlands) at a dose of 10 mg/kg three times weekly via intraperitoneal injection; one group received a recombinant IL-1 receptor antagonist (anakinra; Amgen, Thousand Oaks, CA, USA) given by continuous infusion at a dose of 5 mg/kg per hour using a subcutaneously implanted minipump (Alzet; Durect Corp., Cupertino, CA, USA) as previously described [19]; group 4 received osteoprotegerin (Fc-osteoprotegerin fusion protein; Amgen), which is a blocker of the interaction between receptor activator of nuclear factor-κB (RANK) ligand and RANK, at a dose of 10 mg/kg three times weekly by intraperitoneal injection [20]; and group 5 received phospate-buffered saline (PBS) buffer only. Treatment was started at the stage of early arthritis (week 6) and lasted for 4 weeks. Mice were killed by cervical dislocation at age 10 weeks, blood was drawn by heart puncture, and the hind paws were dislocated for histological and immunohistochemical evaluation. All animal procedures were approved by the local ethics committee.

### Preparation and histological evaluation of decalcified specimen

Left and right hind paws were fixed in 4.5% buffered formalin overnight and then decalcified in 14% EDTA (Sigma, St. Louis, MO, USA; pH adjusted to 7.2 by addition of ammonium hydroxide) at 4°C until the bones were pliable. Serial paraffin sections (2 μm) of the right hind paw were used for the immunohistochemical analyses.

### Immunohistochemistry of phosphorylated MAPKs

For immunohistochemical detection of the phosphorylated forms of ERK, p38MAPKα and JNK, ethanol dehydrated tissue sections were treated with 3% hydrogen peroxide in methanol followed by digestion with proteinase K (25 mg proteinase K in 50 ml PBS) for 5 min at 37°C and blocking with PBS buffer containing 20% rabbit serum for 1 hour. Then, sections were incubated with monoclonal antibodies to the phosphorylated isoforms of ERK-1 and ERK-2 (clone E-4, dilution 1:20), p38MAPKα (clone D-8, dilution 1:5) and JNK (clone G-7, dilution 1:200; all from Santa Cruz Biotechnology, Santa Cruz, CA, USA) at room temperature for 1 hour and for 30 min with biotinylated goat anti-mouse immunoglobulin (Santa Cruz biotechnology). Antibody binding was detected using an ABC complex (for p38MAPKα and ERK: VECTASTAIN@ABC reagent, Vector, Burlingame, CA, USA; for JNK: StreptABComplex/HRP, Dako, Glostrup, Denmark) and 3,3-diaminobenzidine (DAB; Sigma) as chromogen, resulting in brown staining of antigen-expressing cells.

### Cell-specific double labelling experiments

Characterization of cells expressing ERK, p38MAPKα and JNK was performed by double staining using cell-type specific antibodies. After applying the protocol as described above, the slides were incubated with rat anti-mouse monoclonal antibodies against macrophages (F4/80; Serotec Inc., Raleigh, NC, USA; diluted 1:100), T cells (anti-CD3; Novocastra, Newcastle, UK; diluted 1:200) and fibroblasts (Biogenesis, Dorset, UK; diluted 1:40). Thereafter, the sections were incubated by an alkaline phosphatase conjugated rabbit anti-rat immunoglobulin (Dako) and the reaction was visualized using a rat alkaline phosphatase–antialkaline phosphatase complex (Dako), nitroblue tetrazolium (0.25 μg/ml) and 5-bromo-4-chloro-3-indolyl phosphate (0.125 μg/ml). In case of staining for B cells (rat monoclonal antibody against CD45R/B220; BD Biosciences Pharmingen, San Jose, CA, USA; diluted 1:300) the cell-specific antibody was applied first and detected by ABC/DAB with subsequent staining of ERK, p38MAPKα and JNK, and detection with a mouse-specific alkaline phosphatase–antialkaline phosphatase complex system (Dako). Expression of ERK, p38MAPKα and JNK was quantitatively assessed by counting both total numbers of synovial cells and the numbers of positively stained cells in each immunohistochemical staining using a magnification of 200× or by counting positively stained cells per high power field (magnification 400×).

### Immunoblotting

Hind paws from three wild-type and three hTNFtg mice aged 10 weeks were snap frozen in liqid nitrogen and mechanically homogenized at 4°C in buffer containing 20 mmol/l HEPES, 0.4 mol/l NaCl, 1.5 mmol/l MgCl_2_, 1 mmol/l DTT, 1 mmol/l EDTA, 0.1 mmol/l EGTA and 20% glycerol, as well as protease and phosphatase inhibitors (protease and phosphatase inhibitor cocktail, cataolgue numbers P8340 and P2850; Sigma) using an Ultra-Turrax T50 homogenizer (Rose Scientific Ltd., Edmonton, Al, Canada). Tissue extracts were then separated from debris and fat by centrifuging at 13,000 rpm for 15 min. Protein content was measured by Bradford assay and 200 μg tissue protein was subjected to electrophoresis on a 10% SDS polyacrylamide gel followed by transfer onto nitrocellulose membranes. After blocking, the membranes were incubated by antibodies against the phosphorylated as well as total p38MAPKα, ERK and JNK (all antibodies from Cell Signaling, Beverly, MA, USA).

### Statistical analysis

Data are expressed as mean ± standard error of the mean. Expression of ERK, p38MAPKα and JNK in the different therapy groups and cell types was compared by means of Kruskal–Wallis test and Dunn's multiple comparison test.

## Results

### Systemic overexpression of TNF leads to activation of p38MAPKα and ERK pathways in the synovial membrane

**Figure 1:**
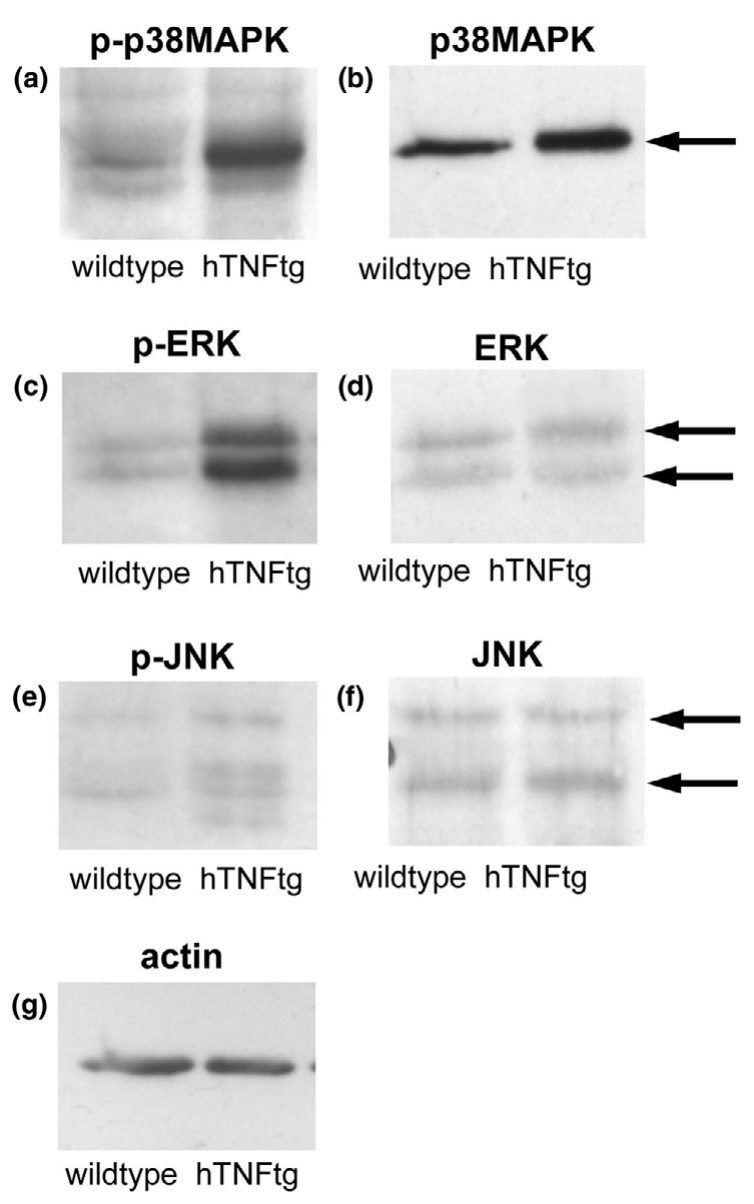
TNF leads to activation of MAPK in arthritic joints of hTNFtg mice. Pooled protein extracts from three normal wild-type mice (left lanes) as well as three arthritic human tumour necrosis factor transgenic (hTNFtg) mice (right lanes) aged 10 weeks were analyzed for the phosphorylated forms of **(a) **p38 mitogen-activated protein kinase (MAPK)α, **(c) **extracellular signal regulated kinase (ERK), and **(e) **c-Jun amino-terminal kinase (JNK) by immunoblotting. In addition, total **(b) **p38MAPKα, **(d) **ERK and **(f) **JNK, as well as **(g) **actin, were analyzed.

**Figure 2:**
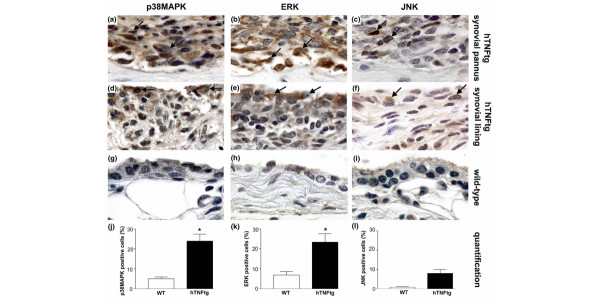
Expression of MAPK in synovial lining and pannus cells of hTNFtg mice and wild-type mice. The phosphorylated forms of **(a,d,g) **p38 mitogen-activated protein kinase (MAPK)α, **(b,e,h) **extracellular signal-regulated kinase (ERK), and **(c,f,i) **c-Jun amino-terminal kinase (JNK) were stained in both the synovial pannus (panels a–c) and the synovial lining layer (panels d–f) of human tumour necrosis factor transgenic (hTNFtg) mice as well as in wild-type mice (panels g–i). p38MAPKα and ERK were abundantly activated in hTNFtg mice but not in wild-type mice (brown staining, black arrows). In contrast, JNK was activated far less frequently and only in a few cells within synovial pannus as well as the synovial lining in hTNFtg mice. In wild-type mice, activation of MAPKs was generally low. Original magnification 1000×. Quantitative analysis showed a significantly higher expression of **(j) **p38MAPKα and **(k) **ERK in hTNFtg mice compared with wild-type mice, but no significant difference in **(l) **JNK activation. Data are expressed as mean ± standard error of the mean. *P < 0.05. WT, wild-type.

To gain an overview of MAPK expression in TNF-mediated arthritis, paw extracts from wild-type and arthritic hTNFtg mice were analyzed for the activated phosphorylated forms of p38MAPKα, ERK and JNK. Paws of hTNFtg mice exhibited marked activation of both p38MAPKα and ERK (Fig. 1a,c) compared with wild-type mice. In contrast, only weakly increased activation of JNK was found (Fig. 1e). Total amounts of p38MAPKα, ERK and JNK were not different among wild-type controls and arthritic hTNFtg mice (Fig. 1b,d,f). To investigate more closely the activation of MAPK by TNF *in vivo*, we histologically assessed joints of hTNFtg mice and wild-type mice for phosphorylated forms of p38MAPKα, ERK and JNK. Synovial inflammatory tissue of hTNFtg mice exhibited widespread activation of p38MAPKα and ERK. Expression of phosphorylated forms of both p38MAPKα and ERK was abundant at sites of destructive synovial pannus but also in the synovial lining layer (Fig. 2a,b,d,e). In contrast, activation of JNK was far less frequent and confined to a few cells within synovial pannus and the synovial lining (Fig. 2c,f). Compared with hTNFtg mice, synovial tissue of wild-type mice exhibited little activation of all three MAPKs (Fig. 2g–i), at most confined to scattered cells in the synovial lining.

We performed a quantitative analysis of MAPK activation, and found p38MAPKα to be activated in 24 ± 4% of synovial cells of hTNFtg mice whereas it was only activated in 5 ± 1% in the synovium of wild-type mice (Fig. 2j). Similarly, activation of ERK was significantly higher in synovial tissue of hTNFtg (23 ± 4%) than wild-type mice (7 ± 2%; Fig. 2k). Activation of JNK was considerably less frequent than each of the two other MAPKs, accounting for 8 ± 2% of cells in the synovial membrane of hTNFtg mice and being virtually absent in wild-type mice (Fig. 2l).

### Macrophages and fibroblasts dominate MAPK activation in the synovial membrane

**Figure 3:**
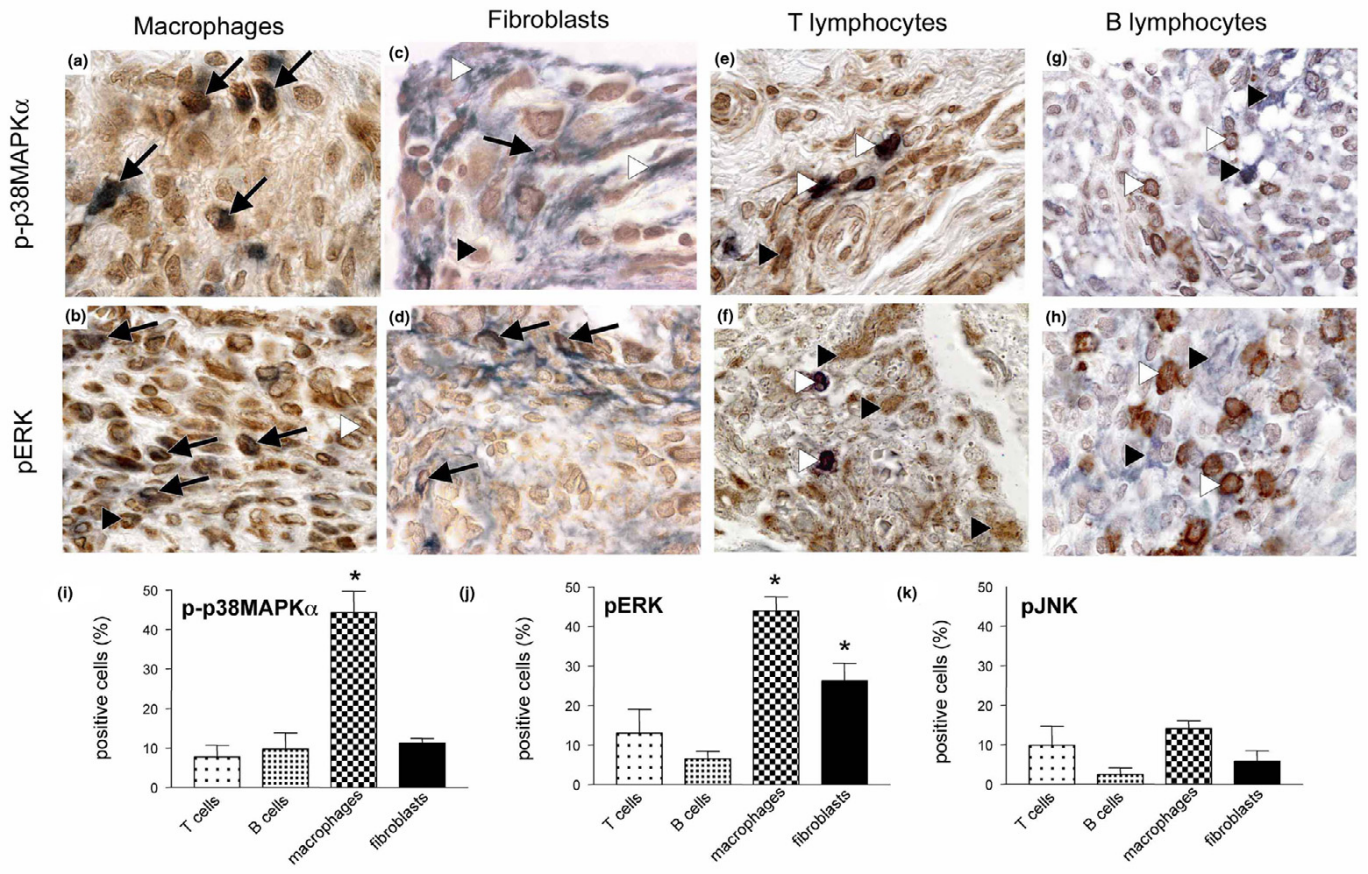
Cell-specific activation of p38MAPKα, ERK and JNK in the inflamed synovial membrane. Microphotographs showing synovial tissue of human tumour necrosis factor transgenic (hTNFtg) mice stained for the phosphorylated forms of p38 mitogen-activated protein kinase (MAPK)α (upper panels) and extracellular signal-regulated kinase (ERK; middle panels), and cell-specific markers for **(a,b) **macrophages, **(c,d) **fibroblasts, **(e,f) **T lymphocytes and **(g,h) **B lymphocytes. p38MAPKα is most frequently present in macrophages (panel a; simultaneous brown and blue staining, black arrows) and less frequently in fibroblasts (panel c; black arrows). Usually, T cells (panel e, white arrowhead) and B cells (panel g; white arrowhead) are negative for activated p38MAPKα (black arrowheads). Activated ERK is present most frequently in macrophages (panel b) and fibroblasts (panel d; simultaneous brown and blue staining, black arrows), whereas it (black arrowheads) is only rarely expressed in T cells (panel g) and B cells (panel h; white arrowheads). Original magnification 1000×. In the lower panels, bars indicate the relative number of cells exhibiting activation of **(i) **p38MAPKα, **(j) **ERK and **(k) **JNK. Analyses were performed for T lymphocytes, B lymphocytes, synovial fibroblasts and macrophages. Activation of p38MAPKα was significantly more frequent in macrophages than in T cells, B cells and fibroblasts (all *P *< 0.05); activation of ERK was significantly more abundant in macrophages and fibroblasts than in T cells and B cells (*P *< 0.05). Data are expressed as mean ± standard error of the mean.

To investigate the cellular expression of the three MAPKs in more detail, we performed immunohistochemical double staining with cell type specific antibodies against macrophages, T lymphocytes, B lymphocytes and fibroblasts. Activation of p38MAPKα was most frequent in macrophages (44 ± 5%; Fig. 3a,i). In contrast, a significantly (*P *< 0.01) lower proportion of T lymphocytes (7 ± 3%), B lymphocytes (9 ± 4%) and fibroblasts (11 ± 1%) exhibited activation of p38MAPKα (Fig. 3c,e,g,i). Similarly, ERK activation was found predominantly in macrophages (43 ± 3%; Fig. 3b,j); however, activation in synovial fibroblasts was also frequently observed (26 ± 4%; Fig. 3d,j). Activation of ERK was significantly (*P *< 0.01) less frequent among T lymphocytes (13 ± 6%) and B lymphocytes (6 ± 2%; Fig. 3f,h,j). As described above, activation of JNK was generally weak. When present, it was found predominately in macrophages, of which 14 ± 2% were positive. Activation of JNK in fibroblasts (6 ± 3%), T lymphocytes (9 ± 5%) and B lymphocytes (3 ± 2%) was generally very low (Fig. 3k).

### TNF but not IL-1 and RANK ligand blockade reduces both p38MAPKα and ERK activation in the inflamed synovial membrane

**Figure 4:**
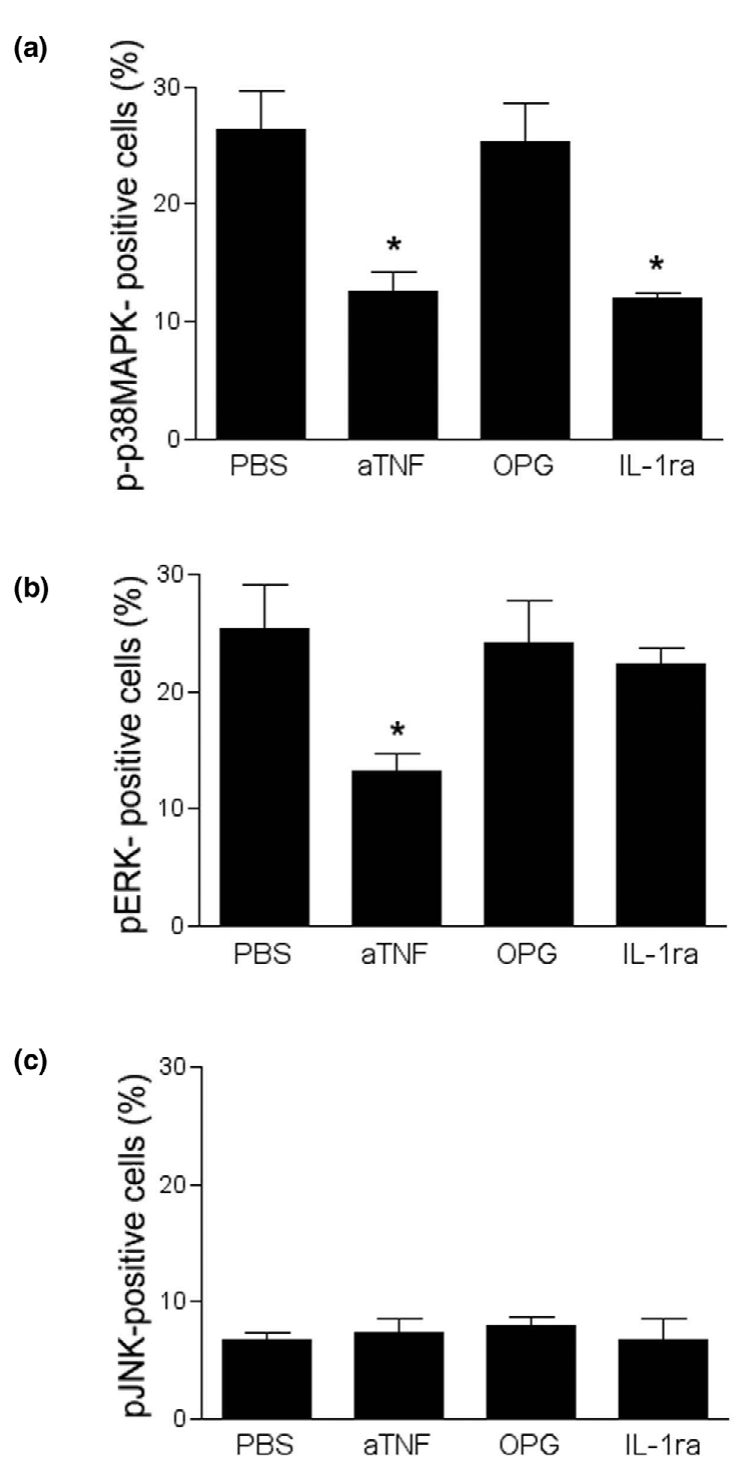
Effects of cytokine blockade on MAPK activation in the inflamed synovial membrane. Bars indicate the percentages of cells expressing the activated forms of **(a) **p38 mitogen-activated protein kinase (MAPK)α, **(b) **extracellular signal-regulated kinase (ERK) and **(c) **c-Jun amino-terminal kinase (JNK) after treatment with vehicle (phosphate-buffered saline [PBS]), anti-tumour necrosis factor (aTNF), osteoprotegerin (OPG) and IL-1 receptor antagonist (IL-1ra). Anti-TNF significantly reduced expression of activated p38MAPKα and ERK; IL-1ra only affected p38MAPKα activation; and OPG led to changed MAPK activation in the synovial membrane. Data are expressed as mean ± standard error of the mean. *P < 0.05.

We next addressed whether cytokine blockade affected increased activation p38MAPKα, ERK and JNK in the inflamed synovial tissue. We compared the activation of these proteins in synovial tissue of hTNFtg mice after systemic inhibition of TNF, IL-1 and RANK ligand. Activation of p38MAPKα was significantly (*P *< 0.05) inhibited by TNF and IL-1 blockade, reducing the fraction of synovial cells exhibiting p38MAPKα activation by 53% and 55%, respectively (Fig. 4a). In contrast, blockade of RANK ligand by osteoprotegerin had no effect on activation of p38MAPKα in synovial tissue. Activation of ERK was significantly affected by TNF blockade only, exhibiting a significant reduction by 48% compared with untreated hTNFtg mice (Fig. 4b). In contrast, both IL-1 and RANK ligand blockade had no effect on ERK activation in the synovial tissue. JNK activation, which was relatively weak compared with the two other signalling molecules, was not altered by any of the three cytokine blockers (Fig. 4c).

## Discussion

In the present study we used hTNFtg mice as an *in vivo *model to define the effects of TNF on MAPK activation the synovial membrane. Cytokine induced signalling through MAPK is considered to be an important mechanism of joint inflammation and represents an interesting option for future antirheumatic therapies [16,17]. We found that TNF predominantly activates p38MAPKα and ERK in the synovial membrane, whereas JNK activation is less common. Furthermore, we were able to demonstrate that macrophages and synovial fibroblasts are the major targets for TNF-induced MAPK induction. Activation of p38MAPKα is clearly dominant in synovial macrophages, whereas activation of ERK is additionally found in synovial fibroblasts. In contrast, TNF-induced activation of MAPK appears not to be critical in lymphocytes. We also showed that cytokine blockade, especially blockade of TNF, effectively interferes with MAPK activation.

TNF is a pluripotent cytokine, which has the potential to induce highly divergent cellular effects. Dependent on the signalling pathway used, TNF can promote cell survival but also programmed cell death, and it is involved in different processes such as inflammation and host defence [11,21,22]. More detailed information on the signalling molecules employed by TNF in chronic inflammation will extend our understanding of how TNF promotes synovitis and may indicate which targeted therapeutics may become feasible extensions of TNF blockade. Because TNF is currently considered a major target of antirheumatic drugs, studies of its role in activating signalling pathways in the synovial membrane deserve further attention. Such studies may be conducted using an *in vivo *disease model based on overexpression of TNF [7]. Although this model has limitations as a model for RA, as is evident from its independence from an autoimmune pathogenesis of arthritis, it nonetheless allows study of synovial changes provoked by a single, well defined trigger, namely TNF.

The data obtained in the present study reveal that induction of synovitis by TNF is accompanied by activation of p38MAPKα and ERK signalling in synovial macrophages and fibroblasts *in vivo*. Earlier *in vitro *studies showed that TNF-mediated cellular effects, including induction of cytokines such as IL-6 and IL-8, as well as the expression of matrix metalloproteinases such as matrix metalloproteinase-13, are dependent on the activation of p38MAPKα and ERK [23,24]. Moreover, p38MAPKα and ERK can both transactivate nuclear factor-κB – a transcription factor known to be essential for inflammation [25]. Taken together, our results support an important role for signalling through p38MAPKα and ERK in mediating the effects of TNF in inflammatory joint disease. Macrophages and synovial fibroblasts appear to be the major targets of TNF-induced MAPK activation, whereas this process is of minor importance in lymphocytes. This cellular pattern of MAPK activation is similar to that observed in human RA, in which p38MAPKα and ERK are mainly activated in macrophages and fibroblasts but not lymphocytes [18].

The observation that TNF leads to activation of p38MAPKα and ERK in the synovial membrane indicates a potential role for pharmacological inhibition of these two MAPKs in blocking the deleterious effects of TNF on the joint. In fact, studies conducted in animal models of arthritis have shown efficacy of small molecule based inhibitors of p38MAPKα in reducing joint inflammation [14,15]. Inhibition of ERK activation has thus far not been applied in inflammatory joint disease but it was used in an experimental model of osteoarthritis [26]. In contrast to the aforementioned kinases, only limited activation of JNK occurs upon stimulation by TNF. Considering the fact that JNK is activated in the synovial membrane of RA [18], this may point to a distinct regulation pattern for JNK in which TNF is not the major player. Recent studies have revealed that JNK activation in synovial cells depends on MEKK-2, an upstream MAPK that is utilized by various growth and differentiation factors such as epidermal growth factor and c-kit [27-30]. This suggests that other proinflammatory mechanisms, which act independently from TNF, may lead to activation of JNK in the synovium. The observation that blockade of JNK reduces structural damage in collagen-induced arthritis – an autoimmune-triggered model of RA that does not exclusively depend on TNF – supports this idea [31,32]. Apparently, such TNF-independent mechanisms are also responsible for the expression of the δ-isoform of p38MAPK in the synovial fibroblast, which is activated by retrotransposable viral sequences termed L1 elements [33].

Currently used cytokine blockers interfere with the binding of the target cytokine with its receptor. As a consequence, the intracellular signalling pathways of the respective cytokine that undergo activation should be blocked or at least inhibited upon use of the cytokine blocker. However, this concept has been poorly investigated. Part of the present study addressed the role of cytokine blockers on TNF-induced MAPK activation. Blockade of TNF significantly reduced activation of both p38MAPKα and ERK in the synovial membrane, indicating that the intracellular effects of TNF can be inhibited. This suggests that anti-TNF therapy reduces key signalling pathways in the synovial membrane, such as the MAPKs, and thereby reduces inflammatory response in the tissue exposed to TNF. Reduction in MAPK activation on cytokine blockade was effective throughout the different cellular compartments and did not significantly change the distrubution of MAPK activation among the various cell types. However, we were unable to reverse MAPK activation with TNF blockade to the level in wild-type mice, suggesting that upregulation of inflammatory mediators downstream of TNF plays a role in synovial MAPK activation. Indeed, inhibition of IL-1 also reduced p38MAPKα activity but not ERK activation, suggesting that at least part of TNF-mediated effects on p38MAPKα are mediated through IL-1. This contributes to the current hypothesis that IL-1 is an important downstream mediator of TNF. It is also in accordance with the observation that p38MAPKα is essential for the proinflammatory action of IL-1 [34]. In contrast, blockade of RANK ligand by osteoprotegerin did not change synovial MAPK activation, which is in good agreement with the observation that blockade of RANK ligand lacks efficacy on synovial inflammation but specifically targets bone degradation in arthritis [19,35,36].

## Conclusion

We show here that TNF leads to activation of two of three MAPK families, p38MAPKα and ERK in the synovial membrane *in vivo*. Inference with the activation of these two MAPKs may therefore be an interesting goal for current and future drug development aimed at inhibiting synovial inflammation.

## Abbreviations

ERK = extracellular signal-regulated kinase; hTNFtg = human tumour necrosis factor transgenic; IL = interleukin; JNK = c-Jun amino-terminal kinase; MAPK = mitogen-activated protein kinase; PBS = phosphate-buffered saline; RA = rheumatoid arthritis; RANK = receptor activator of nuclear factor-κB; TNF = tumour necrosis factor.

## Competing interests

The author(s) declare that they have no competing interests.

## Authors' contributions

BG carried out histological analyses and drafted the manuscript. SH participated in design and coordination of the study. BT carried out histological and statistical analyses. JZ participated in breeding of mice. JSS participated in the design of the study. GS conceived the study, and participated in its design and coordination. All authors read and approved the final manuscript.
